# Discovering Patterns in Brain Signals Using Decision Trees

**DOI:** 10.1155/2016/6391807

**Published:** 2016-09-05

**Authors:** Narusci S. Bastos, Diana F. Adamatti, Cleo Z. Billa

**Affiliations:** Federal University of Rio Grande (FURG), Rio Grande, RS, Brazil

## Abstract

Even with emerging technologies, such as Brain-Computer Interfaces (BCI) systems, understanding how our brains work is a very difficult challenge. So we propose to use a data mining technique to help us in this task. As a case of study, we analyzed the brain's behaviour of blind people and sighted people in a spatial activity. There is a common belief that blind people compensate their lack of vision using the other senses. If an object is given to sighted people and we asked them to identify this object, probably the sense of vision will be the most determinant one. If the same experiment was repeated with blind people, they will have to use other senses to identify the object. In this work, we propose a methodology that uses decision trees (DT) to investigate the difference of how the brains of blind people and people with vision react against a spatial problem. We choose the DT algorithm because it can discover patterns in the brain signal, and its presentation is human interpretable. Our results show that using DT to analyze brain signals can help us to understand the brain's behaviour.

## 1. Introduction

Neuroscience is the scientific study of the nervous system. And with new technologies emerging, such as Brain-Computer Interface (BCI) systems, new discoveries can be made in this area. But analyzing and interpreting data collected from these BCI systems is not an easy job, so software tools have been developed in order to aid scientists in this area.

This work proposes a methodology that uses a data mining technique, called decision tree (DT), to discover patterns in brain signals and presents a model (a decision tree) that is easily interpretable. To show how our methodology works, we use a case scenario where we examine the brain signals of blind people and sighted people in spatial activity.

Blindness is a severe or total change of one or more elementary functions of vision; it affects the ability to perceive color, size, distance, shape, position, or movement in a given space [1]. The expression “visual impairment” refers to the spectrum ranging from blindness to low vision. There are two types of blind people: congenitally blind and acquired blind. The congenitally blind people have the cognitive system based on only 4 senses since birth, without any reference to visual elements, unlike the acquired blind ones, who have to adapt their cognitive system to their new condition [2].

The nervous system is responsible for the reception, storage, and release of information. It is a complex system consisting of various structures and specialized organs with different functions [3]. It can be divided into sensory system, which is responsible for collecting information about the organism and the environment; motor system, which organizes and executes actions; and the associative system. In this work, we focus our study on the sensory system, since it is known that individuals with visual impairments have their orientation capacity compromised [4].

BCI systems are tools (hardware and software) that allow a way of communication between the brain and the computer. They usually capture and process neural activities of the brain, and they do not require other stimuli, such as muscle movements [5]. There are different kinds of BCI; in this work, we used an EEG- (electroencephalogram-) based BCI system to collect brain signals. The EEG records brain electrical activity measured on the surface of the scalp and it is capable of capturing brain activity every millisecond due to a high temporal resolution. Most BCI systems provide only brainwaves and graphics (such as brain maps) tools to analyze the brain activity. In this work, we propose the use of data mining to analyze the brain's behaviour.

Data mining (DM) is the process of extracting or mining knowledge from a large volume of data and there are several algorithms that can be used to discover patterns in a dataset [6]. This work focuses on the use of decision trees for knowledge discovery in a dataset of brain signals of blind (visual impairment) and sighted people during an activity involving spatial abilities in order to discover whether there is a difference in their brain activity.

This work is divided into five sections.  Section 2 presents a theoretical background, such as brain areas and their functions, visual impairment, BCI systems, and data mining.  Section 3 provides the materials and methods we used.  Section 4 presents the obtained results, and finally Section 5 presents conclusions and future works.

## 2. Theoretical Background

### 2.1. Brain Areas and Their Functions

The brain is the main component of the nervous system. It is responsible for all mental operations such as concentration, thinking, learning, and motor control. These capabilities are implemented through neurons, which can currently be explained by neuroscience.

Human brain is divided into two hemispheres, right and left. Initially, there was a belief that there was one dominant hemisphere and the other was dominated. However, this concept has become outdated, and now there is a belief that there are actually two specialized hemispheres. Thus, each hemisphere is responsible for a set of functions that end up working together.

Anatomists usually divide the brain into major regions, called lobes, whose boundaries are not always accurate but transmit an initial idea of regional location. There are five lobes: four external and one internal, located in the lateral sulcus [7]. The four external lobes are the following: frontal lobe, which is located in the forehead; parietal lobe, which is located under the cranial bone with the same name; temporal lobe, which is associated with the tempora; and occipital lobe, which is located in the occipital cranial bone. The fifth lobe, the insula lobe, can only be seen when the lateral sulcus is opened [7, 8]. There are many other structures situated in the central nervous system (CNS), but in this work we investigate only the four visible lobes because the BCI system that we used does not have access to the insula lobe.

Each lobe has specialized functions: the occipital lobe is primarily concerned with the sense of vision; it is divided into multiple distinct visual areas, in which the biggest one is the primary visual cortex. The parietal lobe is partially dedicated to the sense of touch; it is responsible for body sensitivity functions and spatial recognition. The temporal lobe contains the primary auditory cortex; it processes audio data, specific aspects of vision, language understanding, and some aspects of memory. Finally, the frontal lobe is responsible for cognitive actions, memory, and movement [8, 9].

### 2.2. Visual Impairment

The visual impairment, in any degree, compromises a person's ability to orient and move in space with security and independence [10]. So, people with visual impairment or blindness compensate this visions lack of information using other senses: hearing, smell, touch, and taste [11].

### 2.3. Brain-Computer Interface Systems

BCI systems are a set of tools that enable communication between a brain and a computer. The main objective of BCI systems is to provide interaction between a user and an external device, such as computers, switches, or prostheses, using only brain signals. There are different ways to collect brain signals; one of them is to use the electroencephalography (EEG). The EEG is based on detecting brain electrical activity through electrodes applied to the scalp [12].

The signals that are captured by an EEG equipment are the potential differences between regions of the cortex. These electrical signs are generated due to the flow of ions between the different neurons of the brain. When a neuron is activated, it is polarized, generating an action potential that can be propagated to other neurons, provoking a flow of information [13].

The records acquired through the electrodes represent the intensity of brainwaves. They can vary between 0 *μ*V and 200 *μ*V, and they have frequency ranging from 0.3 Hz to 100 Hz. The resulting signal of an EEG shows peaks related to existence of electric activity, indicating a general spatial location of brain activity, because this signal is the sum of the activity of a large number of neurons communicating with each other [14].

#### 2.3.1. Actichamp and Acticap

**Figure 1:**
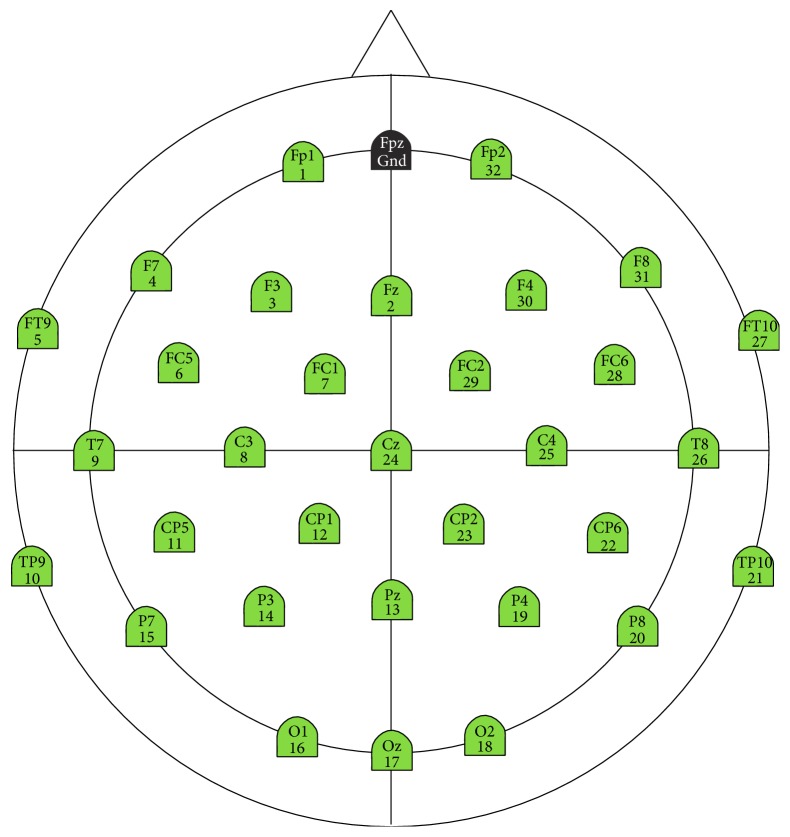
Location of the electrodes in Acticap.

The Actichamp tool is developed by Brain Vision LLC. It is a modular amplification system that incorporates large components for electrophysiological analysis as EEG, event-related brain potentials (ERP), and BCI. It was used in conjunction with Acticap, which is a cap with 32 electrodes, and it is inserted into the scalp of a person. It has the channels of the international standard “10–20.” The Acticap is connected to the Actichamp amplifier, to transmit the signals captured by the electrodes.  Figure 1 shows how the electrodes are distributed throughout the cap.

The locations for each electrode are calculated to be in the intersection of the lines between standard cranium landmarks (see Figure 1). The name of each electrode indicates the region of the brain: FP indicates the prefrontal lobe; F, frontal lobe; T, temporal lobe; C, the central groove; P, parietal lobe; and O, occipital lobe. The number or the second letter identifies the hemispheric location: Z is the zero line in the center of the head; even numbers represent the right hemisphere; odd numbers represent the left hemisphere. The numbers are displayed in ascending order with increasing distance from the center [15, 16].

Table 1 shows the brain areas, the channels that constitute each area, and the abilities of each region.

#### 2.3.2. OpenVibe Software

OpenVibe is a software platform dedicated to designing, testing, and using Brain-Computer Interfaces. The configuration for use with Actichamp is predefined; the software communicates automatically with the signal capture tool. OpenVibe presents a very simple interface, where the user can set through an algorithm (automata) features that meet the needs of the task.

### 2.4. Data Mining

Data mining (DM) is the process of extracting or mining knowledge from a large volume of data. DM involves the study of tasks and techniques, where tasks are a specific class of problems and techniques are the groups of solutions to solve them [6].

Alencar et al. [17] point out that one of the most accepted definitions of data mining by researchers in the field is the one given by Fayyad et al. [18], which states the following: “database knowledge extraction is the process of identifying valid, new, potentially useful and understandable patterns embedded in the data.”

Data mining is one step in a broad process known as Knowledge Discovery in Database (KDD). KDD is the process of finding knowledge in data. In this context, DM is the step of obtaining the information [6].

Descriptive tasks are focused on discovering patterns that describe data in a way that human being can understand. The main descriptive tasks are association rules and clustering. Predictive tasks search for patterns to infer new information about the existing data or to predict the behaviour of new data. The main predictive tasks are classification and regression [6, 19].

The difference between predictive and descriptive methods consists in the fact that descriptive methods do not require a precategorization of records; that is, it is not necessary target in an instance; in predictive methods, the dataset has a predefined target variable and records are categorized in relation to it.

## 3. Related Work

In the last years, a lot of work has been developed using data mining algorithms and, in some of them, these algorithms are used to perform classification tasks in brain signals.

Ishfaque et al. [20] report an experiment with several different classifiers in order to identify if a subject is moving his/her right hand forward or backward or his/her left hand forward or backward, thereby establishing a classification problem of 4 classes. In their experiment, they collect brain signals, using EEG-based BCI, of blindfolded subjects doing random movements of their right and left hands.

Ishfaque et al. [20] collected brain activities from 19 electrodes. The data was processed in the time domain, so their dimensionality was reduced from 19 columns to 5 columns using Principal Component Analysis (PCA). The amplitude of all four classes differentiated in time made the remaining data clearly separable.

After the data was collected, Ishfaque et al. [20] tested different types of classifiers in order to analyze the performance of each one. They use the following classifiers: Artificial Neural Networks (ANN), Linear Discriminant Analysis (LDA), and DT. To evaluate the results, they use a confusion matrix and accuracy percentage (AP). Both measures are used to check the accuracy of the classifiers.

According to the authors in [20], LDA, as expected, divided data linearly and because of that it did not present good classification ratings. On the other hand, ANN and DT reached good classification ratings, with ANN having better results. ANN accuracy percentage was 81.6%, DT accuracy percentage was 75.6%, and LDA accuracy percentage was 24.0%, as we can see in Table 2.

Another work that investigates machine learning algorithms in order to classify brain signals is the one of Wang et al. [21]. They made an experiment where subjects had identified left or right arrows and pressed the corresponding key on the keyboard. They collected two datasets using an EEG-based BCI system.

In the work of Wang et al. [21], they tested the following classification algorithms: LDA, Quadratic Discriminant Analysis (QDA), Kernel Fisher Discriminant (KFD) analysis, Support Vector Machines (SVM), Multilayer Perceptron (MLP), Learning Vector Quantization (LVQ), ANN, K-Nearest Neighbors (KNN), and DT. The unit of measurement for evaluation was the accuracy.

Wang et al. [21] conclude that Gaussian SVM and KNN reached good performance ratings in both datasets, while LVQ, QDA, KDF, and MLP reached the lowest ratings. Linear SVM and LDA presented similar performance. Wang et al. [21] point out that KNN is not commonly used to classify brain signals, but with the appropriate resources extraction and reducing the vector's dimension, KNN can reach good performance ratings.  Table 3 shows the reached accuracy percentage of each classifier in the experiments of Wang et al. [21].

The works of Ishfaque et al. [20] and Wang et al. [21] compare classification algorithms to evaluate which one has better results to classify brain signals in a specific domain. They propose the use of different kinds of classifiers and measure their accuracy. In this work, we propose to use a classification algorithm, specifically DT, to discover patterns of reasoning in the brain. In other words, we do not propose to use DT to classify if the subject is performing a specific action; we are interested in discovering how the brain behaves when a specific task is proposed, and to investigate this brain's behaviour, we used a DT algorithm to discover patterns in the brain signals.

## 4. Methodology

**Figure 2:**
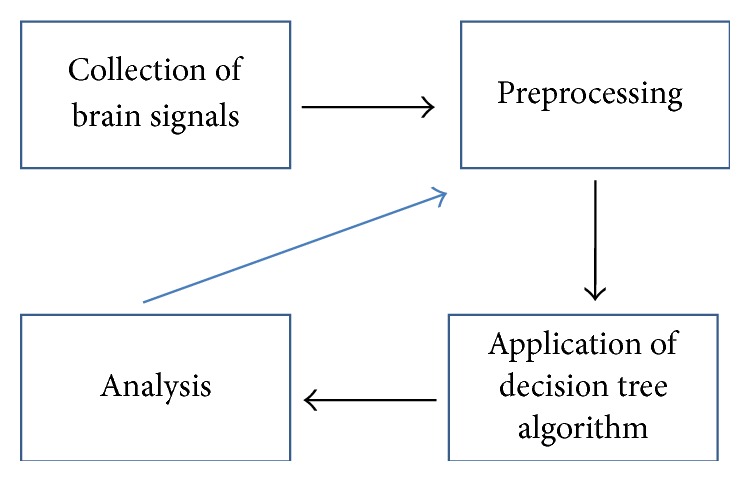
Methodology proposed in this work.

Figure 2 is the flowchart of our methodology. Each box is described in detail in the following subsections.

### 4.1. Collection of Brain Signals

We acquired the brain signals of 4 female individuals: 2 not blind and 2 blind people. The task we gave to them was to identify different 3D solid geometric shapes, in order to stimulate their spacial abilities. In our protocol, we used three objects: ball, cube, and parallelogram. All tests were performed with the approval of the Research Ethics Committee at the Health Area in Brazil, CCAAE: 344172114.3.0000.5324.

Detailed data collection is as follows:

1. The data was collected in a private room, with only the subject and the researchers.
2. The tools used to collect the brain signals were Actichamp and Acticap.
3. Calibration of the equipment: the electrodes have to be stimulated until they showed enough impedance to make it possible to start the collection.
4. The electrodes were connected to an audio recorder and the OpenVibe software was used for the acquisition and monitoring of brain signals.
5. The eyes of the subject were blindfolded.
6. Object one was given to the subject.
7. The subject handled the object and verbalized the name of the object.
8. Steps (6) and (7) were repeated with objects two and three.

**Figure 3:**
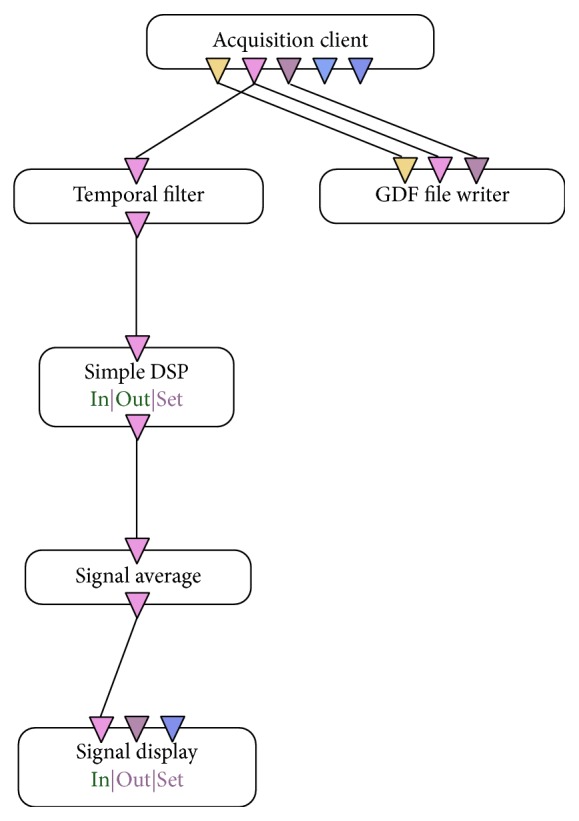
Automata used for the acquisition of brain signals.

Figure 3 shows the OpenVibe's automata used for brain signal acquisition and visual monitoring of them. The automata that follow the left side ending in “signal” display are performed, since the algorithms do not interfere with the signal acquisition. For signal acquisition, only the “acquisition client” and “GDF file writer” are used.

The “acquisition client” waits for data from the EEG and it distributes the signals to the scenario. The algorithm opens a socket to read the experiment information, sign, stimulus, and channels location data sent across the network.

The “GDF file writer” is a function that writes to disk a specific current output in standard file format GDF (Graph Exploration System). This “box” does not allow changes (the user can just inform the file name to be saved).

### 4.2. Preprocessing

The main steps of data preprocessing are

1. conversion from GDF file to CSV file
2. balancing data
3. normalization
4. grouping.

#### 4.2.1. Conversion from GDF File to CSV File

**Figure 4:**
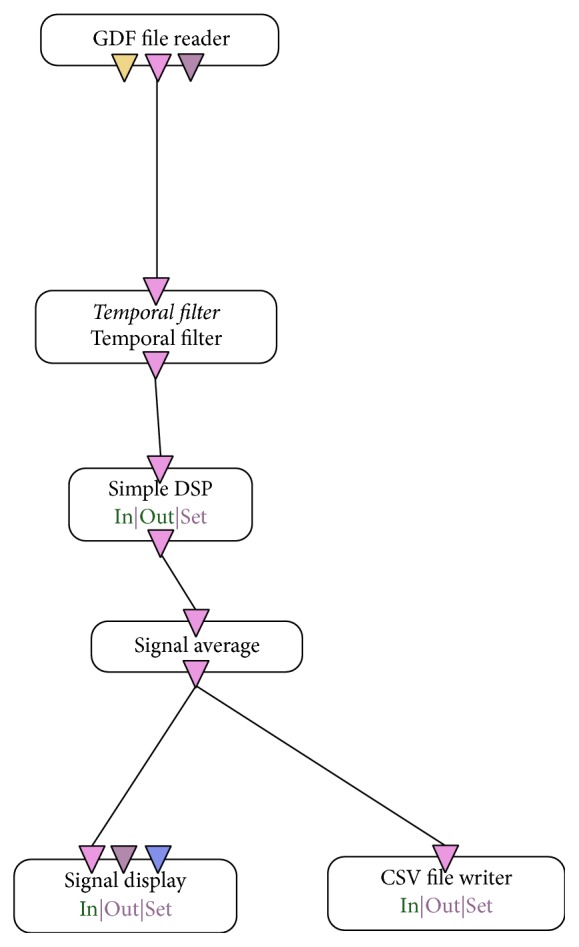
Automata to convert from GDF file to CSV file.

For the conversion from GDF to CSV, it was necessary to create a scenario in OpenVibe software also containing the filters required for analysis of brain signals (Figure 4). This transformation was necessary because the CSV files can be read directly by the Weka data mining tool.

**Figure 5:**
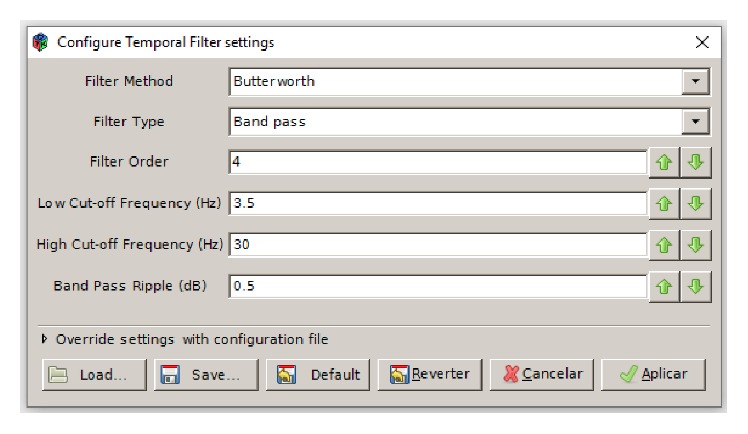
“Temporal filter” settings.

(1) “GDF file reader” has the following configuration:
  (a) Samples per buffer: 32
  (b) Subtracting physical minimum: false
(2) “Temporal filter” is used to filter the input signal (Figure 5).
 The Butterworth filter is designed to introduce a flatter frequency response in the passband. The frequency ranges from 3.5 to 30 Hz, getting the theta, alpha, and beta waves.
(3) “DSP filter” was set to *x∗x* to remove negative signs.
(4) “Signal average” was used to calculate the mean of each input sample and outputs a resulting signal.
(5) “CSV file writer” was used to record the filtered data in a CSV file.
(6) “Signal display” was used for monitoring the data during format conversion.

#### 4.2.2. Balance

As a decision tree algorithm can be influenced by unbalanced data, we had to balance the data. Using the Weka software, we use a random filter to get 40 instances of each class. The number 40 was chosen because it was the number of instances of the class with less instances.

#### 4.2.3. Normalization

The normalization step was done in Weka software, applying the filter “normalized,” which transforms the values of the instances on a scale of 0 to 1.

#### 4.2.4. Grouping

In order to identify temporal patterns, the data were grouped in a period of 1 second (10 instances). For each channel, the highest value was kept. So, at the end, each class had 4 instances, creating a new table with 16 instances.

### 4.3. Application of Decision Tree Algorithm

In this study, we have used the J48 decision tree algorithm, which is a classification algorithm. The J48 tree decision (also called C4.5) is an algorithm that uses the method of divide and conquer to increase the predictive ability of decision trees. In this way, it always uses the best step assessed locally, without worrying if this step will produce the best solution, takes a problem, and divides it into several subproblems, creating subtrees between the root and the leaves. We used the Weka software to execute the J48 algorithm.

The Weka (Waikato Environment for Knowledge Analysis) is a collection of machine learning algorithms for data mining tasks. It was developed by the Department of Computer Science at the University of Waikato, New Zealand [22]. These algorithms can be applied directly or used by Java programs. Weka contains algorithms for preprocessing, classification, regression, clustering, and association rules [19].

### 4.4. Analysis

**Figure 6:**
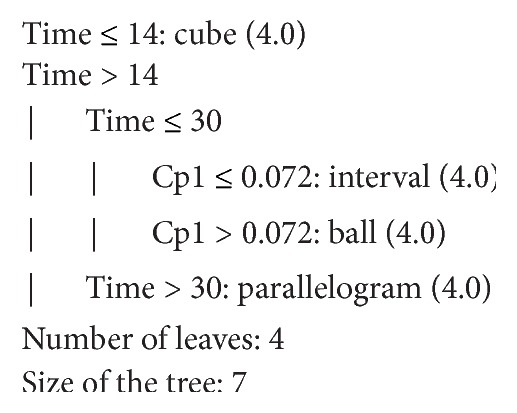
Algorithmic representation of the decision tree.

**Figure 7:**
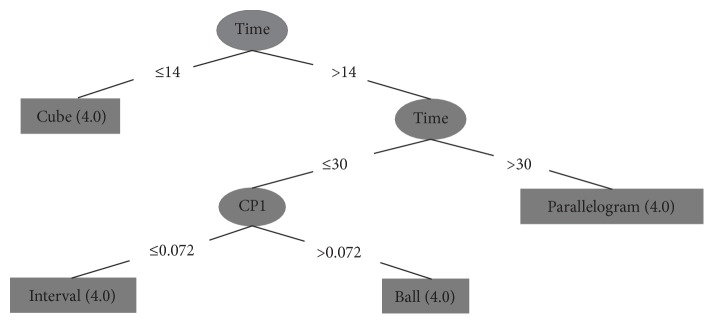
Graphical representation of the decision tree.

In a decision tree, each leaf node receives a class label. The nonterminal nodes, including the root node and other internal nodes, contain attributes test conditions to separate records that have different characteristics [23]. Figures 6 and 7 show an example of trees generated by the J48 algorithm. From the generated tree, we can extract some rules as follows:

1. Time ≤ 14: cube.
2. Time > 14, time ≤ 30, and CP1 ≤ 0.072: interval.
3. Time > 14, time ≤ 30, and CP1 > 0.072: ball.
4. Time > 14 and time > 30: parallelogram.

## 5. Results

During our research, we performed several experiments using the J48 algorithm with different settings. Firstly, the full set of examples was tested. In second place, some electrodes that we do not consider relevant were cut. Thus, instead of 32 channels (all), only 12 channels were used.

**Figure 8:**
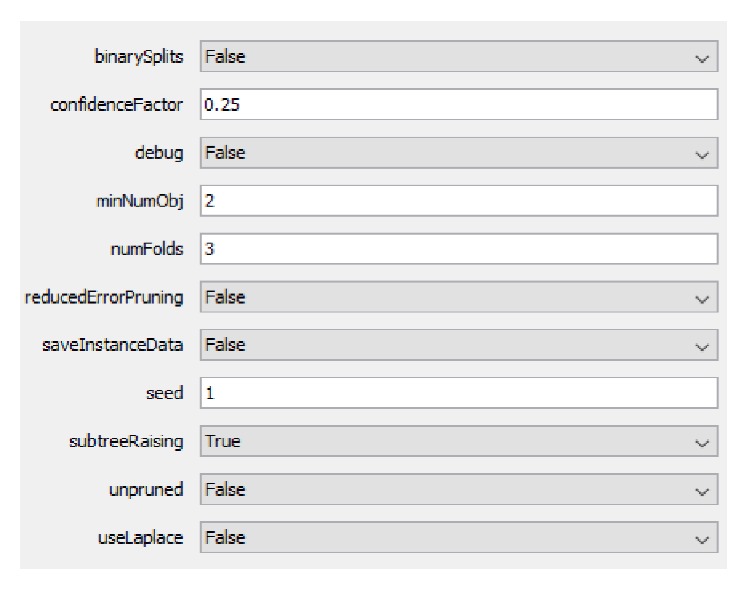
J48 parameters.

Beyond the above-mentioned settings, we also executed tests by varying the minimum number of instances per leaf (minNumObj in Weka), which is a parameter of the J48 algorithm. We tested the values 1%, 5%, and 10% of the total number of instances. The other parameters were set with the default values of Weka, as shown in Figure 8.

In most of these tests, we get good classification ratings, but the generated trees were very large and difficult to analyze. As our goal is not to classify new instances but to use decision trees to discover which areas of the brain had more significant activities, a tree with too many branches is not easy to analyze. We suppose that these trees were getting very large, probably, due to overfitting, since each tree leaf classified very few instances.

**Figure 9:**
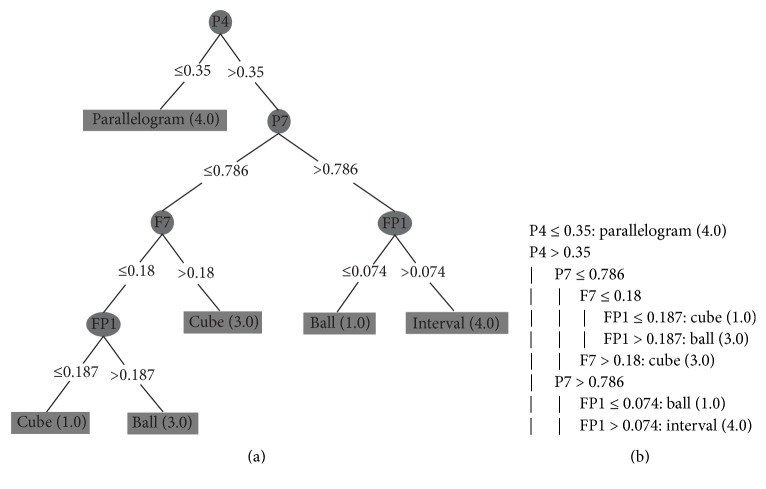
Blind person I: (a) DT's graphical representation and (b) DT's algorithmic representation.

**Figure 10:**
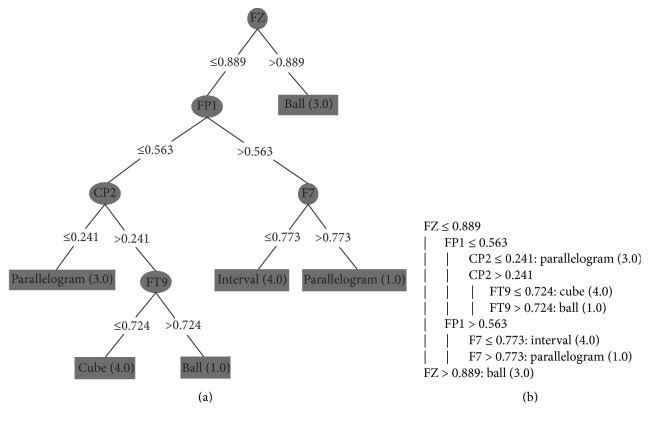
Blind person II: (a) DT's graphical representation and (b) DT's algorithmic representation.

**Figure 11:**
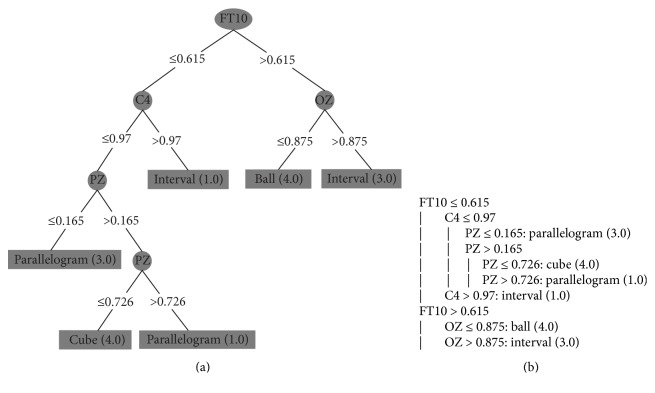
Sighted person I: (a) DT's graphical representation and (b) DT's algorithmic representation.

**Figure 12:**
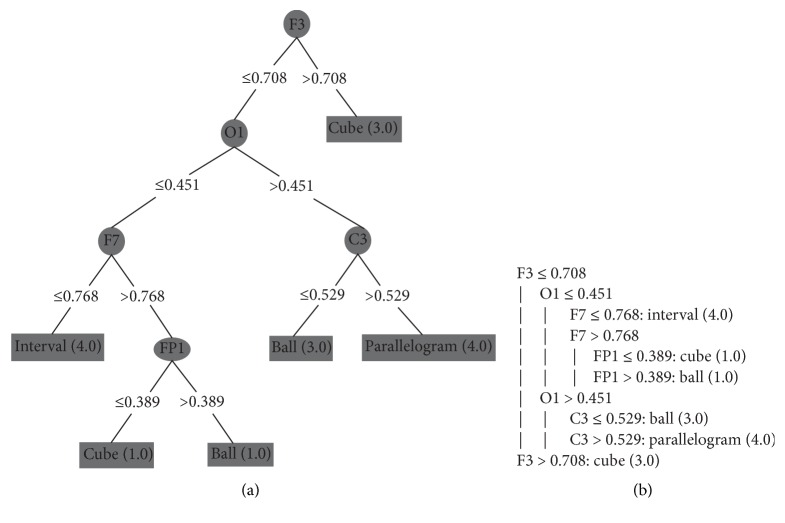
Sighted person II: (a) DT's graphical representation and (b) DT's algorithmic representation.

To avoid this overfitting, we grouped a subset of 10 consecutive instances. Each attribute received the highest value of its set (as mentioned in Section 4.2). So, in these tests, each instance contains the peak of each electrode in a larger period of time, representing in one instance a set with the highest values for each electrode during period of 1 second (approximately). With this configuration, we apply the DT algorithm to each subject's dataset. We executed them with the minimum number of objects set to 1%, because we had a small amount of instances of each class. The generated decision trees are shown in Figures 9, 10, 11, and 12 and they classify 100% correctly all the instances.

Figures 9, 10, 11, and 12 present the decision trees generated by the J48 algorithm, where (a) and (b) are two different ways to represent the same decision tree: (a) is a graphical representation of a tree and (b) is the algorithmic representation. Figures 9 and 10 represent the decision trees of two blind people. And Figures 11 and 12 represent the trees of sighted people.

In the trees of visual impairment individuals (congenital blindness) (Figures 9 and 10), the trees show that the channels presenting the most significant activities were at the parietal lobes (Figure 9: P4, P7; Figure 10: CP2) and at the front lobe (Figure 9: F7, Fp1; Figure 10: Fz, Fp1, FT9, and F7). As we mentioned before (Table 1), the parietal lobe is responsible for coordinating actions that are sensitive to skin, as the sense of touch. The frontal lobe coordinates motor activities, thinking, and speech.

In the trees of sighted individuals (blindfolded), according to Figures 11 and 12, the channels that presented significant activity correspond to the frontal lobe (Figure 11: FT10; Figure 12: F3, F7, and Fp1), parietal lobes (Figure 11: Pz), occipital lobes (Figure 11: Oz; Figure 12: O1), and central lobe (Figure 11: C4; Figure 12: C3). We can notice that, in all tests, blind and sighted individuals showed high activity in the frontal and parietal lobes, where the first is responsible for the organization of the thoughts and the second is responsible for the sense of touch. However, sighted people showed significant activity in the occipital lobe, which is responsible for the sense of vision. In blind people, this area was not activated, because the J48 algorithm considered the values of the occipital lobe's electrodes insignificant for the generated model.

## 6. Conclusion

This work analyzed four individuals, including two not blind people and two people with congenital visual impairment, in order to analyze brain activity during a spatial activity. For this analysis, we collect the brain signals with Actichamp tool and process these data with the Weka software to data mining. We choose the J48 data mining classification technique because it generates decision trees that are easy to analyze.

Based on the resulting decision trees, we can observe that sighted people had significant activity in the occipital lobe, which is responsible for the sense of vision, even when they are blindfolded. We suppose that this happened because they accessed the visual memory to aid them to identify the objects. However, blind people showed no significant activity in the occipital lobe in the model created by the J48 algorithm. Therefore, our experience suggests that the brains of blind people and people with normal vision have different ways of carrying out spatial activities, even if they are placed in the same situation (people with vision were blindfolded).

As future works, we intend to expand the study with a larger number of people. We also intend to apply the methodology in other neuroscience studies, since DT can be used to discover patterns and understand brain signals in many different kinds of problems.

## Figures and Tables

**Table 1:** Brain region, electrodes, and proprietary functions.

Brain region	Electrode	Proprietary functions
Frontal lobe	Fp1, Fp2, Fz, F7, F3, Fz, F4, F8, FC5, FC1, FC2, FC6, FT9, FT10.	Executive functions (management of cognitive/emotional resources on a given task)

Temporal lobe	T7, TP9, T8, TP10.	Perception of biological motion

Parietal lobe	P7, P3, Pz, P4, P8.	Somatosensory perception, spatial representations, and tactile perceptions

Occipital lobe	O1, Oz, O2.	View images (including during a dialogue)

**Table 2:** Classifiers accuracy percentage [20].

Classifier	PA
ANN	81.6%
LDA	24.0%
DT	75.6%

**Table 3:** Percentage of the accuracy of the classifiers in different datasets [21].

Classifier	Dataset I PA	Dataset II PA
LDA	82.86%	84%
QDA	78.57%	79%
KFD	80.71%	81%
Linear SVM	82.86%	82%
Gaussian SVM	84.29%	84%
MLP	80.71%	81%
LVQ	77.86%	80%
*K*-NN	84.29%	83%
DT	82.14%	86%
